# Useful road maps: studying *Drosophila* larva’s central nervous system with the help of connectomics

**DOI:** 10.1016/j.conb.2020.09.008

**Published:** 2020-12

**Authors:** Claire Eschbach, Marta Zlatic

**Affiliations:** 1 Department of Zoology, University of Cambridge, United Kingdom; 2 MRC Laboratory of Molecular Biology, United Kingdom

## Abstract

The larva of *Drosophila melanogaster* is emerging as a powerful model system for comprehensive brain-wide understanding of the circuit implementation of neural computations. With an unprecedented amount of tools in hand, including synaptic-resolution connectomics, whole-brain imaging, and genetic tools for selective targeting of single neuron types, it is possible to dissect which circuits and computations are at work behind behaviors that have an interesting level of complexity. Here we present some of the recent advances regarding multisensory integration, learning, and action selection in *Drosophila* larva.

## Highlights

• *Drosophila* larva enables combining comprehensive synapse resolution connectivity maps with cellular-resolution activity maps and behavior maps.
• This approach provides a way to elucidate neural implementation of universal brain computations such as multisensory integration, learning, and action-selection.
• Early multisensory convergence recruits specific sensorimotor loops for specific actions.
• Higher-order brain areas integrate modalities in a more centralized way and produce high-order, valence-like signals for goal-oriented behavior.

**Current Opinion in Neurobiology** 2020, **65**:129–137

This review comes from a themed issue on **Whole-brain interactions between neural circuits**

Edited by **Larry Abbott** and **Karel Svoboda**

For a complete overview see the Issue and the Editorial

Available online 23rd November 2020

**https://doi.org/10.1016/j.conb.2020.09.008**

0959-4388/© 2020 The Author(s). Published by Elsevier Ltd. This is an open access article under the CC BY license (http://creativecommons.org/licenses/by/4.0/).

## Introduction

Ability to sense, act, remember, or anticipate emerges from the way the nervous system is organized into networks that allow signals to flow, interact, and change. Nerve cell types and numbers vary across different organisms, but many neuronal computations and behaviors appear to be done in a similar way across mammals and insects. For example, odor signals are processed via two parallel high-order pathways differing in representation and plasticity [1,2]. Feeding circuit is formed of multilayered loops linking external/enteric sensory inputs to motor/secretory outputs [3,4]. Dopaminergic neurons encoding reinforcement of different valences project onto spatially distinct associative regions [5].

Given the phylogenetic distance between insects and mammals (half a billion year), the fact that similar circuit solutions for complex problems have been conserved or reinvented through evolution points towards some fundamental principles linking structure and function in the central nervous system (CNS).

**Figure 1:**
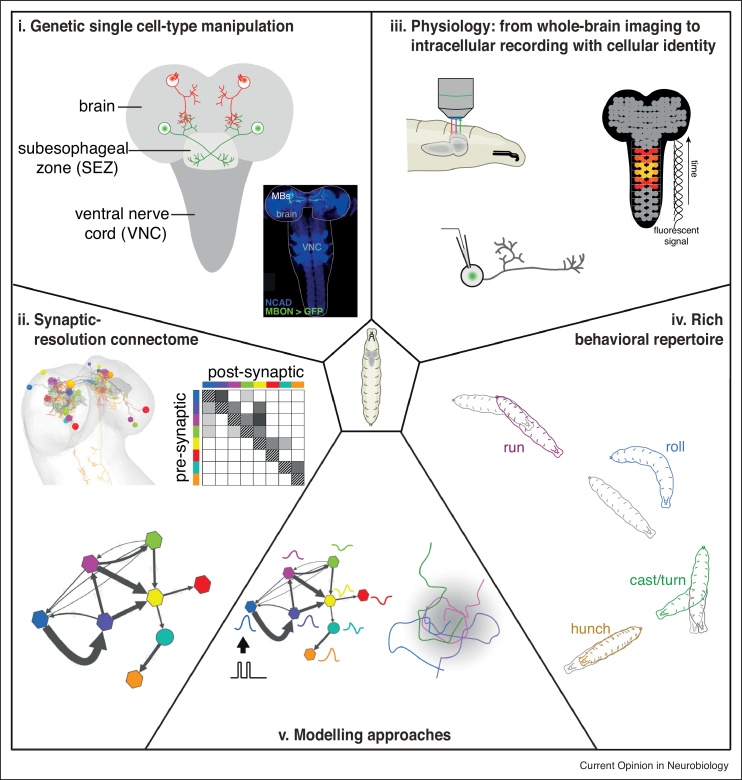
Approaches for studying neural circuit in *Drosophila* larva. ***i.*** Drosophila larva beneficiates from a comprehensive genetic toolkit for selective targeting of uniquely identified neuron types, often a single pair of left-right homologous neurons [21,22]. *Left*, schematic of the tripartite organization of the larval CNS: brain, SEZ and VNC. *Right*, confocal image of a CNS immunostained against n-cadherin (*blue*) and GFP expressed in a single pair of MB output neurons (*green*). ***ii.*** Synaptic resolution connectome. A full CNS imaged with EM has been reconstructed; the connectivity of many neural circuits at synaptic resolution is now known. Quantitative studies have shown that strong connections are symmetrical between left and right side [7] and conserved across individuals and larval stages [19]. ***iii.*** Accessibility to physiological activity: *in vivo* imaging [26, 27, 28, 29, 30, 31], *ex vivo* whole-brain imaging [23], intracellular recordings of uniquely identified neurons that can be selectively labelled by GFP [8,9,18,77]. ***iv.*** Substantial behavioral repertoire: discrete actions such as run, stop, head cast, turn, hunch, backup, roll can be automatically tracked and categorized. ***v.*** Modelling approaches. Thanks to the few number of larval neurons (*ca*. 15,000 including 2500 brain neurons), reconstructed connectivity can be implemented in an artificial neural network [8,16,18]. Behavioral hallmarks can also be reproduced by an agent-based model [52,56,67].

Here we review recently described circuits in the CNS of the larva of *Drosophila melanogaster.* The insect CNS comprises a brain, a subesophageal zone (SEZ), and a ventral nerve cord (VNC, Figure 1**i**). This tripartite organization is similar to the forebrain/cerebellum, brainstem, and spinal cord of vertebrates. The relatively small CNS of the *Drosophila* larva as a model offers a number of advantages for studying the circuit implementation of neural computations in a comprehensive way (Figure 1).

## Studying neural circuits in *Drosophila* larva

The early larval central nervous system contains fewer (*ca*. 15,000) and smaller neurons compared to the adult *Drosophila* making it amenable to relatively rapid electron microscopy imaging and circuit reconstruction with synaptic resolution [6] (Figure 1**ii**). So far, circuits for somatosensory processing [6, 7, 8] and motor programming [9, 10, 11] in the VNC, feeding [3] and neuromodulation [12] in the SEZ, as well as first-order sensory [14,15], and higher-order associative centers [16, 17, 18] in the brain have been reconstructed with synaptic resolution in the same EM volume of a first-instar (*i.e.* early larval stage) nervous system, and comprehensive reconstruction of the CNS is within reach. The reproducibility of this type of data has been tested by Gerhard *et al.* [19], who compared portions of nociceptive circuits in an early (first-instar) and a later (third-instar) stage larvae, and found that the fraction of total synaptic input associated with defined pre-synaptic partner is maintained despite a five-fold change in size. In many cases, the knowledge about connectivity could be augmented with immunohistology against neurotransmitters. This has allowed the identification of various types of circuit motifs [8,14, 15, 16,18].

The connectome alone is not sufficient for understanding circuit mechanisms [20], but it provides a necessary roadmap for mechanistic studies. In complement, *Drosophila* larva is amenable to a large variety of functional studies (Figure 1**i,iii**). With multiple genetic tools for selective targeting and manipulating individual cell types [21,22], functional connectivity between neurons can be tested by combining optogenetic activation of presynaptic neurons with *e.g.* electrophysiological recording [8,9,18] or calcium imaging of postsynaptic neurons [6,10,13,18]. Imaging the activity of motoneurons can also be used as a proxy for behavior and allows the visualization of fictive actions [23, 24, 25]. Due to the transparency of its body wall, imaging in living animals has recently been developed in immobilized [26,27] as well as in moving animals [27, 28, 29, 30, 31].

In addition, the larva has a rich behavioral repertoire, exhibiting a range of distinct actions and sequences [6,8,32, 33, 34, 35, 36, 37, 38, 39, 40, 41, 42] (Figure 1**iv**) and is capable of robust associative learning [16, 17, 18,43, 44, 45, 46, 47, 48, 49]. Automatic tracking of individual larvae and behavioral categorization greatly facilitates relating the structure of larval circuits to their function [6,8,13,31, 32, 33, 34, 35, 36, 37,39,49,50]. High-throughput behavioral inactivation and activation screens allow identifying neurons promoting or repressing specific actions or computations [8,33,36,41,50,51].

Finally, these approaches are often combined with modelling that can generate testable predictions about the possible roles of specific circuit motifs in behavior [6,8,11,16,18,37,52,56] (Figure 1**v**).

## Circuits for multisensory integration, learning, and action-selection

As described in other organisms [57], specific sensory modalities act jointly early on in signal processing, at the first or second-order neuron, to build a meaningful representation of a stimulus. Later in processing, at higher-order brain regions, more sensory modalities can be combined to keep track of environmental variability. Circuit analyses in *Drosophila* larva are starting to elucidate the way in which all dimensions of multisensory experiences are integrated for appropriate action selection.

### Multisensory integration at early stages

In the VNC, local sensorimotor loops influence crucial aspects of locomotion and distinct types of responses to various somatosensory stimuli [6,8,27,30,34,38,58, 59, 60, 61]. Among them, nocifensive behaviors rely on multidendritic nociceptive neurons [6,38,60,61]. Depending on the type of threat, larvae can respond to nociceptive stimulation by accelerating forward [34,39,62], crawling backward [34], or rolling sideways [6,38,60,61], the latter being the most vigorous nocifensive response (Figures 1**iv**, 2 ). Neural activation and inactivation screens revealed neurons necessary and/or sufficient for nocifensive behaviors [6,33,34,38,39,61,63]. Connectome reconstruction unraveled how sensory inputs target these bottleneck neurons [6, 7, 8,19,34,61].

**Figure 2:**
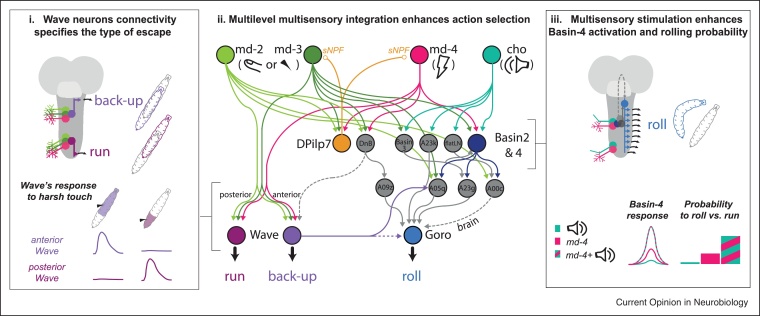
Circuits for nocifensive behavior in larva. Sensorimotor circuits for escape response selection to a nociceptive stimulus have been particularly well studied in *Drosophila* larva. The particular nocifensive behavior depends on the type of threat: predator attack, noxious heat or harsh touch can evoke rolling response [6,38,60,61], instead weaker and less threatening stimuli can evoke fast forward crawling or back-up [34,39,62]. The nature of the response relies on the type of somatosensory neurons co-activated with the polymodal larval nociceptors (multidendritic class IV ‘md-4’, *magenta*). Components of the circuits reconstructed downstream of the nociceptive neurons [6,61] and of the main somatosensory neurons (chordotonal ‘cho’, ‘md-2’ and ‘md-3’, *greens*) [7,8,34] are depicted. Circles indicate pairs or class of neurons, plain lines are direct connections, dotted lines indirect ones, all reconstructed in the same EM volume. ***i.*** ‘Wave’ neurons repeatedly tiling the VNC integrate spatially defined multimodal inputs to promote either run or back-up in response to, respectively, posterior or anterior harsh touch stimulation [34]. These neurons also indirectly contact the roll-promoting neuron ‘Goro’ [6,34]. ***ii.*** Multiple levels of multisensory integration occur during the selection of rolling: both early in sensory processing, and at more downstream nodes in the network [6, 7, 8,19,34,60,61]. Modelling shows that multiple levels of multisensory integration enhances action selection [6]. ***iii.*** ‘Basin 4’ super-additively integrates ‘cho’ and md-4’ inputs. Increased Basin-4 activation by multisensory cues, in turn increases the likelihood of rolling behavior, through Goro activation [6].

Forward or backward escapes have been shown to rely on the homologous segmentally repeated ‘Wave’ neurons [34] that integrate two types of somatosensory inputs: touch sensors and nociceptors. Importantly, Wave neurons in posterior segments induce forward escape, whereas the ‘Wave’ neurons located in the anterior segments induce backward escape (Figure 2**i**). EM reconstruction revealed Wave neurons in all segments receive synaptic input from nociceptive and touch-sensing neurons, but they have different output targets in different segments: the anterior Wave neurons synapse onto circuits in anterior segments that promote backward crawling, whereas the Wave neurons target posterior segments and promote forward crawling. Combining EM reconstruction with functional studies therefore revealed the way in which homologous neurons integrate and target different partners in different regions of the nervous system to mediate opposite behaviors.

The most vigorous and energetically costly rolling escape is elicited in response to predator attack [38]. Presenting mechanosensory cues with nociceptive ones facilitates rolling [6,61], likely because it better approximates predator attack which also stimulates multiple senses. Rolling is mediated by a command-like ‘Goro’ neuron that receives indirect functional inputs from both mechanosensory and nociceptive sensory neurons [6,61]. EM reconstructions of circuits downstream of mechanosensory and nociceptive neurons and upstream of the Goro neurons elucidated precisely where and how the information from these distinct sensory modalities is integrated. This revealed that mechanosensory and nociceptive information converges early on in the sensory processing hierarchy onto first-order multisensory interneurons that integrate the information superadditively [6] **(**Figure 2**ii).** Multiple interneurons, gathering slightly different somatosensory modalities, relay multisensory nociceptive inputs to Goro and are sufficient to evoke rolling [6,61]. Additionally, later stages of multisensory integration at higher-order nodes enhance action selection [6]. Furthermore, when activated in combination with nociceptive neurons, touch-sensing neurons integrate the multiple mechanosensory inputs through sNPF feedback release and facilitate rolling [60]. Thus, knowing both how the different nodes of circuits are connected and their functional properties provided a mechanistic insight into the way in which nocifensive behaviors are selected (Figure 2).

### Higher-order integration: learning and value coding

Larval behavior is plastic and adapts to experience (review in [47]). Mushroom Bodies (MB) in the larval brain are necessary to form associative memory [43]. This memory is expressed by changing navigation towards a cue (*e.g.* an odor) that has been associated with a reward (*e.g.* sucrose) or a punishment (*e.g.* quinine).

**Figure 3:**
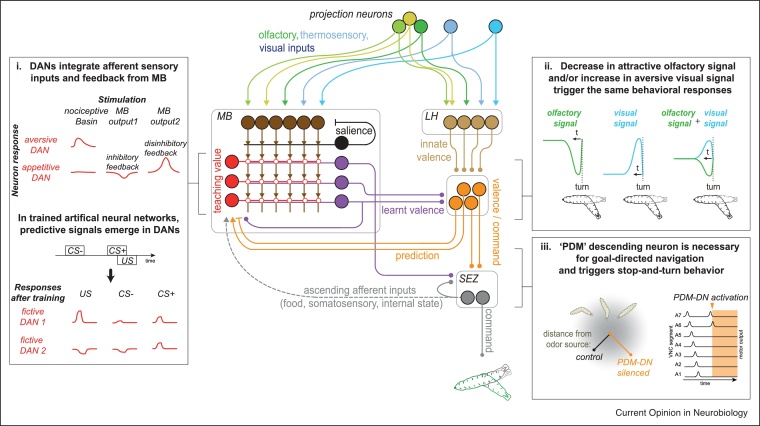
Circuits for high-order integration and value computation. In the larval brain the MB and the LH receive convergent sensory inputs from olfactory, thermosensory and visual projection neurons (*green and light blue*) [16,14,15]. The LH neurons (*light brown*) are assumed to parse the information according to innate valence. The MB Kenyon cells (*dark brown*) are thought to decorrelate sensory signals via highly divergent connections, expose them to reinforcement-gated plasticity (*red open circles*), and generate learnt valence signals (*purple*). MB is characterized by a recurrent architecture: a GABAergic neuron (*black*) gathers signals at the KCs axons and feeds back onto the KCs dendrites [16,17]; in addition, KCs signal back to the teaching neurons (mostly DANs, *red*) [16,49] and so do the MB output neurons (*purple and orange feedbacks*) [16,18]. These multilayered paths provide neural substrates for integrating prediction to the teaching signals, in addition to other inputs from the SEZ, for adaptive memory update (See ***Box i*** [18]). Further down the circuit, MB and LH are likely integrated for valence signals that can be used as instructive signal for navigation (*orange*, See ***Box ii*** [54] and ***iii*** [35]). The SEZ (*grey*) transforms more [16,35] or less [3] integrated signals into behavior. ***i.****Top panel,* the experimental exploration of the responses of some teaching DANs, combining calcium transient and optogenetic stimulations, confirms that DANs integrate external (multisensory nociceptive neurons Basin) and memory-related (MB outputs neurons) inputs combining the recording of cell activity and optogenetic stimulations. *Bottom panel*, In an artificial neural network incorporating the connectivity of the MB and the response tuning of the DANs, discriminative signal for conditioned stimuli that predict (CS+) or not (CS-) the unconditioned stimulus (US) emerges in the DANs after associative training [18]. ***ii.*** Reverse-correlation experiments [13,53, 54, 55] link olfactory and visual inputs to navigation [54]. Under fictive odor stimulation (the optogenetic activation of receptors for attractive odors continuously varying in intensity), redirecting turns are initiated by a decrease in the signal, while under real visual stimulation (aversive blue light varying in intensity) turns are initiated when the signal increases. The sensorimotor transformation estimated for the combination of inputs suggests a linear integration of olfactory and visual inputs, probably readable at the level of LH output. ***iii.*** PDM-DN, a neuron reconstructed in EM, downstream of LH neurons and descending to the SEZ, is necessary for navigating towards an odor source [35]. The optogenetic activation of this neuron triggers stop and redirection in crawling animals by stopping the wave of body contraction that goes through the body and shutting off the activity of the corresponding motoneurons.

MBs in insects translate rich sensory representation into a low dimension signal relevant for behavior and encoded by the population of MB output neurons (MBONs; [64]) (Figure 3). EM reconstruction of the comprehensive set of 223 intrinsic MB neurons, called Kenyon cells (KCs), in a first-instar larva and all of their pre- and postsynaptic partners revealed the detailed synaptic-resolution architecture of the learning circuit and uncovered a number of unexpected circuit motifs. Most Kenyon cells were found to integrate random combinations of inputs from olfactory projection neurons, but a subset received stereotyped inputs from single projection neurons [16]. Combining the connectome with modelling revealed that this distribution results in enhanced contrast between cues encoded by the KCs, improving performance of a model output neuron on a discrimination task [16]. In addition, as in many other organisms [5], larval dopaminergic neurons (DANs) carry reinforcing signal which lead to appetitive or aversive memory formation (reviewed in [47]). DANs target different regions of the KC axons and gate synaptic plasticity [65] between a subset of KCs activated by a cue and the output neurons extending their dendrites in this region of the MB. The minimal microcircuit composed of KCs-to-MBONs synapses modulated by DANs is sufficient to explain how cues-elicited behavior changes upon associative trials [16,44]. EM revealed additionally that KCs reciprocally synapse onto DANs [16]. Consistent with this, activation of KCs alone (*i.e.* by sensory cues without reinforcement) can lead to plasticity [48]. Whether the KC-to-DAN synapse is subject to plasticity is an exciting open question.

What do DANs encode? Of the 8 DANs projecting onto the MB, 4 are necessary and/or sufficient to form sugar memory [17,46]. Three others are sufficient to form an aversive memory and we found they respond to somatosensory stimulations [18] (Figure 3**i**) Thus, food-related signal from the SEZ and somatosensory signal from the VNC seem translated into dopaminergic signal onto the MB, reminiscent to appetitive and aversive reinforcement encoded by different DANs in the striatum of rodents [5]. EM reconstruction of the circuits upstream of DANs is starting to provide insights into the way in which teaching signals that drive learning are computed. A comprehensive reconstruction of all neurons (109) presynaptic to the DANs (or other modulatory neurons, [16]) [18], revealed that 7 are MBONs [16] and 60 are directly or indirectly postsynaptic to MBONs [18]. In total, the mono- and polysynaptic feedback from MB represent more than 50% of the synaptic inputs received by DANs. Artificial network constrained by the reconstructed larval MB connectivity revealed that these recurrent signals improve performance and flexibility of learning tasks [18]. Strikingly, the responses of the artificial DANs to the different associative cues changed over the course of training, consistent with prediction signals formulated in theories of reinforcement learning and with findings in mammalian DANs (reviewed in [66]). Whether such adaptive responses also naturally emerge during learning in behaving larvae is still an open question.

The many layers of recurrence in the MBs [16, 17, 18,48] and the fact that KCs receive inputs from multiple modalities - olfactory, visual, thermosensory, gustatory [16] (Figure 3)- raise the question of the nature of the signal after MB processing. Many MBONs interact [16] and a few of them integrate inputs from multiple MBONs from compartments of opposite valence [16, 17, 18]. These MBONs are well poised to collect and compute the overall result of MB processing. The way in which the MBON signals are combined with LH outputs that signal innate valence and used to guide action selection is still an open question. Agent-based modelling suggests that a fully centralized system combining all value inputs into a unified value code would reproduce larval navigation accurately [52,56,67] On the other hand, the artificial neural network constrained by the connectome found the coding of predicted value can be represented within the numerous feedback neurons in a distributed way [18], although how this signal is built and used for economic decisions is not yet fully understood. With its few neurons and low redundancy, the brain of *Drosophila* larva is a model of choice to combine more experimental and theoretical approaches and deepen our understanding of these mechanisms.

## Action selection

*Drosophila* larvae can generate many exclusive actions. Studies are beginning to elucidate the way in which multisensory inputs, higher-order valence signals, and context are used for action selection. In recent years progress has been made in understanding the circuits that mediate the selection of distinct types of escape responses in response to threatening somatosensory stimuli: roll, fast crawl, turn, back-up, and hunch. The selection of the most vigorous escape, roll, is enhanced by integrating nociceptive with mechanosensory inputs [6,61] (Figure 2). So far, many circuit motifs involved in the selection of these actions and their organization into sequences have been identified in the VNC. For example, reciprocally connected inhibitory interneurons mediate behavioral choice between hunch and turn in response to an air-puff [8], lateral disinhibition promotes sequence transitions between these actions, and specialized local feedback disinhibition provides positive feedback that stabilizes a behavior and prevents reversals to the preceding one. The combination of these interconnected circuit motifs can implement both behavior selection and the organization of behaviors into a sequence. Interestingly the connectome reveals that descending neurons from the brain and SEZ synapse onto many of the VNC interneurons involved in somatosensory responses [6,8]. The way in which brain circuits bias somatosensory choices is an exciting open question for the future.

A second major action selection paradigm in the larva is the choice whether to crawl or turn and which way to turn when navigating gradients of aversive or repulsive cues. The alternation between runs (forward crawl events), stops, and turns can be done without brain inputs [68] but their transitions based on sensory inputs rely on the brain (besides likely modalities detected by VNC sensory neurons, [8,68]). These choices require the computation of the value of the cue, which is done based on both innate and learnt valences. Ongoing reconstruction of neurons downstream MB and LH will inform about the way in which innate and learnt values are integrated. In addition, different modalities converge onto the same pattern of navigation responses [13,37,40,41,53]. Different sensory inputs have been shown to be translated into turn action following the same signal transformation function [54] (Figure 3**ii**). It is therefore possible that all modalities converge onto a common center involved in computing an overall integrated value of a cue and guiding navigation. This interpretation also fits the convergence of projection neurons of various modalities onto the two brain structures MB [16] and LH [14,15] (Figure 3). To comprehensively characterize the circuits that underlie navigational decisions several screens have been conducted for neurons that contribute to these behaviors [50,69, 70, 71]. Possible bottleneck targets of navigational circuits are the descending neurons, such as the ‘PDM-DN’ which can trigger stop in a deterministic way [35] (Figure 3**iii**). This command neuron has been located in the SEZ [35] while other command neurons are located in the VNC (for roll [6]; for backup [34]). Likely, the VNC and SEZ regions are thus the last steps where conflict for different types of actions may be resolved. Reconstructing the full pathways for different behavioral drives all the way from a comprehensive set of sensory inputs to a comprehensive set of command neurons will allow identifying their sites of interactions and potential conflict resolution; this may result in refining our views of the processes defined as action selection or decision [8,36].

## Conclusion

Providing a precise roadmap with the connectome, *Drosophila* larva helps formulate and test new hypotheses about the way in which neural circuits implement fundamental computations such as multisensory integration, learning, value computation, and action-selection.

Future research will include richer behavioral situations (*e.g*. [42,72,73]), *in vivo* recording of whole-brain activity [23, 24, 25], modelling approaches (*e.g.* [8,11,16,18,52]). In parallel, whole-brain RNAseq reveals genes expressed in individual neurons [74,75] that might be essential for these computations. The comparatively small size enables rapid reconstruction of connectomes from multiple individuals (*e.g.* [76]) and opens doors to an exciting new area of experimental connectomics to address questions about the structural correlates of specific memory traces, individual differences in circuits that underlie distinct personality traces, and discovering the effects of various mutants on the circuit architecture.

## Conflict of interest

The authors declare no conflict of interest.
